# Synthesis of silver nanoparticles using a modified Tollens’ method in conjunction with phytochemicals and assessment of their antimicrobial activity

**DOI:** 10.7717/peerj.6413

**Published:** 2019-02-08

**Authors:** Muna A. AbuDalo, Ismaeel R. Al-Mheidat, Alham W. Al-Shurafat, Colleen Grinham, Vinka Oyanedel-Craver

**Affiliations:** 1 Chemistry Department, Faculty of Science and Arts, Jordan University of Science and Technology, Irbid, Jordan; 2 Department of Civil Engineering, Faculty of Engineering, Jordan University of Science and Technology, Irbid, Jordan; 3 Department of Civil and Environmental Engineering, University of Rhode Island, Kingston, RI, USA

**Keywords:** Silver nanoparticles (AgNPs), Tollens’ method, Olive leaves extract (OLE), Rosemary leaves extract (RLE), Antimicrobial effect

## Abstract

**Background:**

Silver nanoparticles (AgNPs) have attracted great attention due to their outstanding electrical, optical, magnetic, catalytic, and antimicrobial properties. However, there is a need for alternative production methods that use less toxic precursors and reduce their undesirable by-products. Phyto-extracts from the leaves of olive and rosemary plants can be used as reducing agents and (in conjunction with Tollens’ reagent) can even enhance AgNP antimicrobial activity.

**Methods:**

Conditions for the proposed hybrid synthesis method were optimized for olive leaf extracts (OLEs) and rosemary leaf extracts (RLEs). The resultant AgNPs were characterized using UV–visible spectroscopy, an environmental scanning electron microscope, and Dynamic Light Scattering analysis. An atomic absorption spectrophotometer was used to measure AgNP concentration. Fourier transform infrared spectroscopy (FTIR) was used to determine the specific functional groups responsible for the reduction of both silver nitrate and capping agents in the leaf extract. Additionally, the antimicrobial properties of the synthesized AgNPs were assessed against Gram-negative bacteria (*Escherichia coli* and *Salmonella enterica*) and Gram-positive bacteria (*Staphylococcus aureus*), by using both the Kirby–Bauer and broth microdilution methods on Mueller–Hinton (MH) agar plates.

**Results and Discussion:**

A simple, feasible, and rapid method has been successfully developed for silver nanoparticle synthesis by reducing Tollens’ reagent using leaf extracts from olive and rosemary plants (widely available in Jordan). Scanning electron microscopy images showed that the method produces AgNPs with a spherical shape and average core sizes of 45 ± 2 and 38 ± 3 nm for OLE and RLE, respectively. A negative zeta potential (ζ) of −43.15 ± 3.65 mV for OLE-AgNPs and −33.65 ± 2.88mV for RLE-AgNPs proved the stability of silver nanoparticles. FTIR spectra for AgNPs and leaf extracts indicated that the compounds present in the leaf extracts play an important role in the coating/capping of synthesized nanoparticles. The manufactured AgNPs exhibited an antibacterial effect against *Escherichia* coli and *Staphylococcus aureus* with minimum inhibitory concentrations (MIC) of 9.38 and 4.69 μl/ml for OLE-AgNPs and RLE-AgNPs, respectively. The MIC for *Salmonella enterica* were 18.75 μl/ml for both OLE-AgNPs and RLE-AgNPs. Furthermore, our results indicated that the RLE-AgNPs exhibited a stronger antibacterial effect than OLE-AgNPs against different bacteria species. These results contribute to the body of knowledge on nanoparticle production using plant-mediated synthesis and performance. They also offer insights into the potential for scaling up this production process for commercial implementation.

## Introduction

Silver nanoparticles (AgNPs) have attracted great attention due to their outstanding electrical, optical, magnetic, catalytic, and antimicrobial properties (Rai, Yadav & Gade, 2009). In 2015, Vance and coworkers redeveloped the nanomaterials consumer products inventory and listed 1,814 nano-based consumer products from 622 companies within 32 countries—a 28% increase over the 2010 inventory. Almost half of the products (762, or 42% of the total) were intended for health and fitness applications. Moreover, AgNPs were the most frequently used nanomaterial (435 products, or 24%), due to their antimicrobial properties (Vance et al., 2015).

Silver nanoparticles are produced using a wide variety of physical and chemical methods. Most physical methods require high energy consumption, a large space, and/or lengthy time periods. This is due to the need to achieve thermal stability while raising the environmental temperature around the source material in the tube furnace to ensure stable operating temperatures. On the other hand, common chemical methods utilize hazardous reducing chemicals, such as sodium borohydride, hydrazine, or hydrogen, that can have adverse effects on the environment and human health (Sharma, Yngard & Lin, 2009; Abou El-Nour et al., 2010).

Chemical methods involve the reduction of silver salts with a reductant such as citrate acid and a solvent like sodium borohydride. Additionally, a stabilizer is needed to prevent agglomeration of the nanoparticles (Huang & Yang, 2004). Since the mid-1990s, greener methods for producing nanoparticles have been sought (Murray, Norris & Bawendi, 1993; Trindade & O’Brien, 1996; Raveendran, Fu & Wallen, 2003).

Green synthesis encompasses the use of less toxic precursors, a low number of reagents, and benign solvents such as water at close to room temperature—with the expectation of fewer byproducts and waste streams as compared with conventional processes (Lu & Ozcan, 2015; Wong & Karn, 2012). Some proposed green methods comprise the use of mixed-valence polyoxometallates, organic materials (especially polysaccharides), and enzymes and biological organisms as reducing agents, as well as solvents and stabilizers (Sharma, Yngard & Lin, 2009).

Plant-mediated synthesis is one of the most common approaches used in green synthesis: extracts from various plant components, such as leaves and roots, are used as reducing and stabilizing agents (Ahmed et al., 2016b). This process is faster and more benign than conventional methodologies, and can be carried out at room temperature and pressure (Mittal, Chisti & Banerjee, 2013). Furthermore, plant-mediated synthesized AgNPs are expected to be stable, cost-effective, and safe, particularly for human therapeutic use (Sharma, Yngard & Lin, 2009).

Plants that have been effectively used for Ag-NPs synthesis are: pine, persimmon, gingko, magnolia, platanus (Song & Kim, 2009), *Cinnamon zeylanicum* (Sathishkumar et al., 2009), *Mentha piperita* (Lamiaceae) (Mubarak Ali et al., 2011), olive (Khalil et al., 2013), maple (Vivekanandhan et al., 2014), *Euprenolepis procera* (Asgary et al., 2016), and Aloe vera (Tippayawat et al., 2016). The most commonly reported biomolecules responsible for the reduction of precursor and stabilization of nanoparticles are metabolites such as alkaloids, phenolic compounds, terpenoids, and water-soluble co-enzymes (Mittal, Chisti & Banerjee, 2013).

Tollens’ synthesis method using Tollens’ reagent [Ag(NH_3_)_2_]^+^ as a source of Ag^+^ and aldehyde as a reducing agent, produces AgNPs with a controlled size in a one-step process (Yin et al., 2002). On the other hand, in a modified Tollens’ procedure, Ag^+^ ions are reduced by saccharides in the presence of ammonia, yielding silver nanoparticle films (50–200 nm), silver hydrosols (20–50 nm), and AgNPs of different shapes (Kvítek et al., 2005). In this green synthesis technique, the size and morphology of AgNPs were controlled by changing the concentration of ammonia and the nature of the reducing agent. In addition, AgNPs with controllable sizes were also synthesized by the reduction of [Ag(NH_3_)_2_]^+^ with glucose, galactose, maltose, and lactose (Panáček et al., 2006). To increase AgNPs stability, sodium dodecyl sulfate, polyoxyethylene sorbitanemonooleate (Tween 80), and polyvinylpyrrolidone (PVP 360) were used as stabilizing and capping agents (Kvítek et al., 2008; Soukupová et al., 2008).

This work presents the development of a hybrid synthesis method in which rosemary leaf extract (RLE) and olive leaf extract (OLE) were used instead of saccharides to reduce the Tollens’ reagent Ag(NH_3_)_2_^+^ (aq) into AgNPs. OLE and RLE have been used effectively to reduce silver salts directly into nanoparticles that display antimicrobial properties (Shrivastava et al., 2007; Awwad, Salem & Abdeen, 2012; Khalil et al., 2013; Sulaiman et al., 2013). In this research, however, a new reduction approach using a known nanosuspensions stabilizer (PVP) was developed for AgNP synthesis using olive and RLEs in conjunction with Tollens’ reagent. This resulted in a greener synthesis that exhibited adequate antimicrobial properties. Finally, this approach was expected to increase the replicability of the nanoparticles produced in terms of size and antimicrobial properties (Kvítek et al., 2008; Sharma, Yngard & Lin, 2009).

To the knowledge of the authors, no previous study has used this approach for nanoparticle fabrication. Olive (*Olea europaea*) and rosemary (*Rosmarinus officinalis*) were selected because of their ubiquity, economic efficiency, and well-documented nutritional and medicinal applications (Khalil et al., 2013; Pereira et al., 2007; Shelef, Naglik & Bogen, 1980). The olive tree is the most important fruit tree in Jordan, covering about 72% of the total planted area and 36% of the total cultivated area in the country. Between 1991 and 2006, the amount of land devoted to olive cultivation in Jordan quadrupled (Al-Shdiefat, El-Habbab & Al-Sha’er, 2006). With 20 million olive trees supporting 180,000 families, Jordan also ranks eighth in the world among olive-producing nations (The Jordan Times, 2015). Therefore, olive and rosemary can support local AgNPs production by the proposed synthesis process.

## Materials and Methods

### Materials

Silver nitrate (Fischer Scientific, Guangzhou, China, 99.8% analytical reagent grade), polyvinylpyrrolidone (ACROS, Morris Plains, NJ, USA, MW = 58,000, PVP), nitric acid (Merck, Darmstadt, Germany, 69%), nutrient agar, nutrient broth, ammonium hydroxide aqueous solution (Tedia, Fairfield, OH, USA, 25% w/w, ACS grade), and sodium hydroxide pellets (Merck, Darmstadt, Germany, 99%) were used without any further purification. Deionized (DI) water was used for all experiments. Lastly, all glassware was periodically washed with diluted nitric acid (25%) and then dried in a hot-air oven overnight at 40 °C.

Antibacterial tests were then performed using three representative pathogenic species obtained from Princess Haya Biotechnology Center, Jordan University of Science and Technology (JUST) (Irbid, Jordan.): one Gram-positive strain of *Staphylococcus aureus* (ATCC 25923) and two Gram-negative strains of *Escherichia coli* (ATCC 12900) and *Salmonella enterica* (CIP 104220).

### OLE and RLE preparation

Leaves of olive and rosemary were collected in June 2014 from the campus of the JUST in Irbid, Jordan. The collected leaves were transported to the laboratory and left to dry at room temperature (25–30 °C) for 10 days, following the procedure described by Dipankar & Murugan (2012). The leaves were then washed, and subsequently dried in a hot-air oven at 40 °C for 5 days to reduce the loss of the leaves’ constituents, as recommended by Pessoa et al. (2007). The dried leaves were then pulverized into a very fine powder by grinder and stored at 30 °C. The extract was prepared by adding 0.1 g plant powder to 100 ml heated water on a hot plate without stirring, and then leaving the mixture to boil for 10 min to obtain an extract of 0.1 wt% concentration. After that, extracts were cooled to 30 °C and the supernatant was slowly filtered with 0.45 μm polyamide membranes (Sartorius Biolab products; Sartorius AG, Göttingen, Germany) via a pump-filter apparatus to remove any remaining solid residues. The plant leaf extracts (PLEs) were kept at −4 °C to be used later.

### AgNPs Synthesis via Tollens’ method

Silver nanoparticles were prepared using the well-known Tollens’ method. OLEs and RLEs, rather than saccharides, were used as reducing agents (Kvítek et al., 2008) to reduce the Tollens’ reagent Ag(NH_3_)_2_^+^ (aq) into AgNPs.

In addition to the previously prepared extracts, stock solutions of AgNO_3_ (10^−3^ M), sodium hydroxide (1.25 × 10^−2^ M), and PVP (8.4 × 10^−5^ M) were prepared. To prepare AgNP dispersions, a tin-foiled 250 ml Erlenmeyer flask container was used as a reaction vessel and placed on a stirrer plate while a syringe pump apparatus was fitted to feed the vessel with the reducing agent (PLEs). Initially, 12.5 ml AgNO_3_ stock solution was added to the vessel at a stirring speed of 600–700 rpm. Then 38.5 μl of ammonia solution was added dropwise, followed by 12.5 ml of PVP stock solution. Finally, 25 ml of 1:1 PLE:NaOH mixture was slowly added to the vessel at a rate of 75 ml/h using an automatic dosing syringe through a plastic slip. The synthesized AgNP suspension was then ultrafiltrated using a 10 kDa nominal molecular weight cut-off membrane via ultrafiltration stirred cell (Model 8200; Millipore, Burlington, MA, USA, NMWCO:10,000) for concentration, purification, and pH adjustment. Any nitrate, PVP, Ag^+^, or PLE not bounded to the nanoparticles was therefore removed from the solution. The ultrafiltration process consumed up to 600 ml of DI water for each sample, in order to achieve a final purified concentrate of 50 ml in volume. The AgNP nanosuspension was kept in tin-foiled covered containers at 4 °C for a period of 6 months for further characterization and bactericidal experiments.

### Instrumentation for characterization

Optical properties of the prepared AgNP nanosuspension were determined using a UV–Vis spectrophotometer (UV-2550; Shimadzu, Kyoto, Japan). The shape and size of the AgNPs produced were identified using an environmental scanning electron microscope (ESEM) Quanta 450 FEG-USA/EEU that operated at an electron gun power of 30 kV. In order to obtain the ESEM images, the synthesized AgNP dispersions were sonicated for 10 min in an ultrasonic bath (100 watts) that generates ultrasonic waves at a 35 kHz frequency (Ultrasonic LC20H; Elma, Singen, Germany). Then the sonicated dispersions were diluted (1:250,000) and re-sonicated for 30 min. After that, a drop of this dilute nanosuspension was placed on an ESEM pin stub specimen mount for measurement. Average silver core diameter was calculated by averaging 50 particles from the ESEM image. Average hydrodynamic diameter (h_d_), size distribution, polydispersity index (PDI) and zeta potential (ζ) were determined by dynamic light scattering analysis (DLS) using a Malvern Zetasizer (Nano-ZS; Malvern Instruments Ltd., Worcestershire, UK). Measurements were determined three times for each sample, and uncertainties are given as standard deviations. Raw data were subsequently correlated to the mean hydrodynamic size by cumulants analysis (*Z*-average mean), according to ISO 22412:2017 (ISO, 2017). The dispersions were sonicated for 30 min using a 200 watt ultrasonic bath (Jeio Tech, Geumcheon-gu, Korea). No dilution was required before measurement. Fourier Transform Infrared spectra were obtained using a Spectrophotometer (IR_Affinity, Shimadzu, Kyoto, Japan) using potassium bromide pellets at 1:10 dilutions and in the ranges between 400 and 4,000 cm^−1^. Fourier transform infrared spectroscopy (FTIR) measurements were carried out to identify representative functional groups of possible molecules on the surface of the nanoparticles.

### AgNPs synthesis conditions

The effect of the reaction conditions such as PLE concentration, temperature, and pH was evaluated by varying one parameter while the others remained constant; the tested parameters and ranges are shown in Table 1. The UV–Vis spectra were determined in triplicate.

**Table 1:** Synthesis conditions that was investigated using the modified Tollens’ method and PLEs.

PLE concentration (mg/l)	Temperature (°C)	pH
20	80	7.0
100	80	7.0
250	80	7.0
250	20	7.0
250	40	7.0
250	80	7.0
250	80	3.0
250	80	5.0
250	80	6.0
250	80	7.0
250	80	11.0

### Evaluation of AgNPs yield

The OLE-AgNP and RLE-AgNP dispersions were analyzed for their remaining Ag^+^ concentration using a Shimadzu AA-6200 atomic absorption spectrophotometer (AAS). The nanosuspensions were centrifuged at a maximum relative centrifugal force of 4,185×*g* (6,000 rpm) for 15min (Z 200 A; HERMLE Labortechnik, Wehingen, Germany). The centrifuge separates the AgNPs at the bottom of the centrifuge tube, leaving the remaining Ag^+^ in the supernatant. After proper dilution, the obtained supernatants were analyzed. The difference in Ag^+^ concentration between the supernatant of the AgNP suspension and the standard AgNO_3_ stock solution thus represents the amount of silver transformed to AgNPs (Singhal et al., 2011; Raffi et al., 2010).

### Antibacterial susceptibility experiments

Three measures of bacterial growth and viability were used to evaluate the antimicrobial properties of the synthesized AgNPs: The broth microdilution method was used to determine the minimum inhibition concentration (MIC) and minimum bactericidal concentration (MBC), whereas the Kirby–Bauer method was used to assess the sensitivity of bacteria to nanoparticles.

A stock of the AgNP suspension was prepared at a concentration of 100 mg/l and, as recommended by Clinical and Laboratory Standards Institute (CLSI) guidelines (Barry et al., 1999), was tested by broth microdilution using a 96-well microplate. Twofold dilutions of the synthesized AgNPs (e.g., 100, 50, 25, 12.5, 6.25, and 3.125 μg/ml) were prepared in a Mueller–Hinton broth using a 96-well microtitration plate (microdilution), in triplicate. Each well was inoculated with 50 μl of the respective bacterial suspension (of Mueller–Hinton broth) and the concentration was matched against a 0.5 McFarland Standard to obtain a concentration of 1 × 10^6^ CFU/ml. After well-mixing, the inoculated 96-well microtitration plate was incubated at 37 °C for 24 h. Both the MIC and MBC were then detected during this incubation period. Mueller–Hinton broth was used as a negative control.

The Kirby–Bauer method (Bauer et al., 1966) was used to determine bacterial susceptibility to OLE-AgNPs and RLE-AgNPs. A triplicate of 100 μl of the respective bacterial suspension (of Mueller–Hinton broth) with a turbidity of 1 × 10^5^ CFU/ml were spread out on MH agar plates. The cultured MH agar on each plate was perforated with holes (wells) using a sterilized glass tube and labeled appropriately. Each well was filled with 50 μl of either OLE-AgNPs or RLE-AgNPs, and MH agar plates were incubated at 37 °C for 24 h. Finally, the zones of inhibition (ZI) were measured and reported. In all tests, silver nitrate was used as a positive control and a blank agar plate was incubated to detect any contamination that might have occurred during testing.

## Results and Discussion

### Optimum AgNPs synthesis

**Figure 1:**
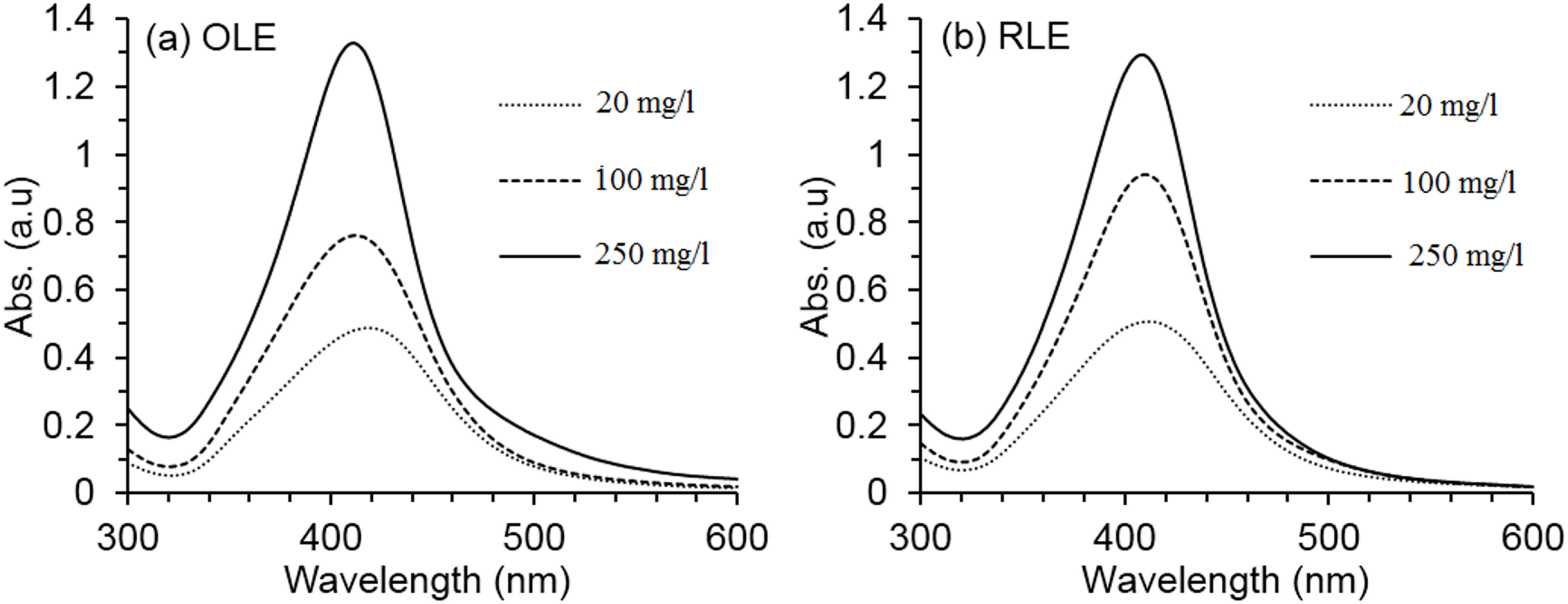
Average AgNPs UV–vis spectra at different PLE concentrations. The synthesis reaction conditions are fixed at temperature 80 °C and pH 7 ± 0.2 for both (A) OLE and (B) RLE.

Optical spectra shown in Fig. 1 confirmed AgNP formation at all PLE concentrations by the detection of the peak of absorption between 410 and 420 nm. This range is characteristic of AgNP spectra, due to excitation of surface plasmon vibration. Resultant colors depend on the particle type, size, morphology, and solvent chemical composition (Bhui et al., 2009; Hao et al., 2004). The color of our synthesized dispersions ranged from brownish-yellow to deep browns, which is also an indicator of AgNP formation (Huang & Xu, 2010). The synthesis depended on the PLE concentration, since absorbance intensity increased by 65% (OLE) and 61% (RLE) by increasing PLE concentration from 20 to 250 mg/l. For both PLEs, the highest concentration tested provided the narrowest absorbance spectra, which indicates a more monodisperse nanosuspension (Bhui et al., 2009). Therefore, the 250 mg/l PLE concentration was selected to investigate the effects of temperature and pH on the AgNP synthesis.

**Figure 2:**
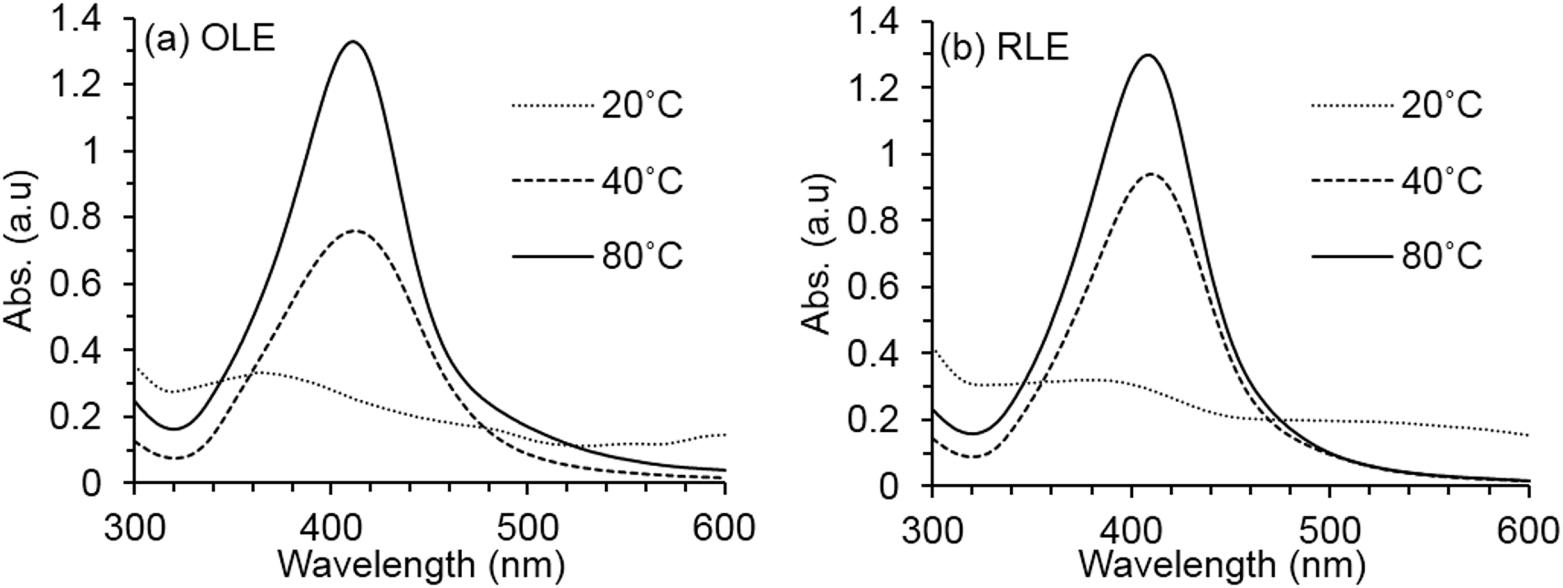
Average AgNPs UV–vis spectra at different synthesis temperature. The synthesis reaction conditions are fixed at PLE concentration of 250 mg/l and pH of 7 ± 0.2 for both (A) OLE and (B) RLE. Measurements were performed in triplicates.

As shown in Fig. 2, the UV–VIS absorbance intensity increased sharply as temperatures rose from 20 to 80 °C for 81% (OLE) and 78% (RLE). Such results illustrate the dependence of the synthesis process on temperature. Therefore, our results suggest that the simultaneous use of Tollens’ reagent and PLEs is effective only at high temperatures (i.e., up to 80 °C). As the temperature increases, the reaction rate increases, and more Ag^+^ is consumed for the formation of nuclei that grow into controlled nanoparticles (Yong & Beom, 2009). Therefore, the temperature of 80 °C was fixed for the next pH effect investigation.

**Figure 3:**
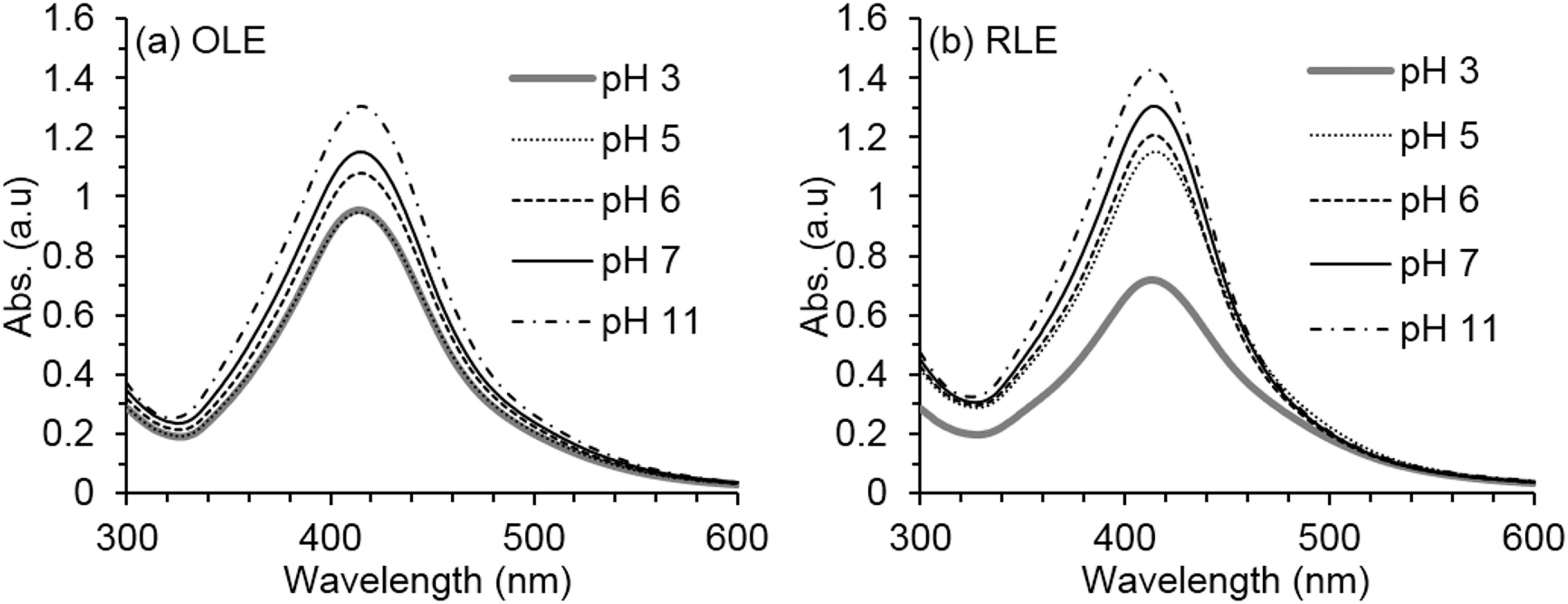
Average AgNPs UV–vis spectra at different synthesis pH. The synthesis reaction conditions are fixed at PLE concentration of 250 mg/l and temperature of 80 °C for both (A) OLE and (B) RLE. Measurements were performed in triplicates.

Favorable pH basic conditions were shown for the reduction process using both OLE and RLE extracts (Fig. 3) with an increase in absorbance intensity from pH 2 to 11 with 27% (OLE) and 50% (RLE). This may be due to the PLE organic compounds, specifically those with carbonyl functional groups which can act as reducing agents only under basic conditions. Higher pH values increased the number of functional groups that were available to bind with silver ions and therefore increased the production of AgNPs. Under our testing conditions, pH 11 ± 0.2 produced the highest yields in terms of nanoparticles production as shown by the UV–VIS spectra. These findings are similar to Khalil et al. (2013), after using OLE as a reducing and stabilizing agent. However, it was decided that reducing the pH during the ultrafiltration process to 7 ± 0.3 is important to the selected three bacteria strains in the antibacterial susceptibility experiments (the optimum pH of the three species ranges between 7.4 and 7.6). Neutral pH is expected to reduce the AgNP agglomeration that was found to be connected to basicity (Veerasamy et al., 2011).

**Figure 4:**
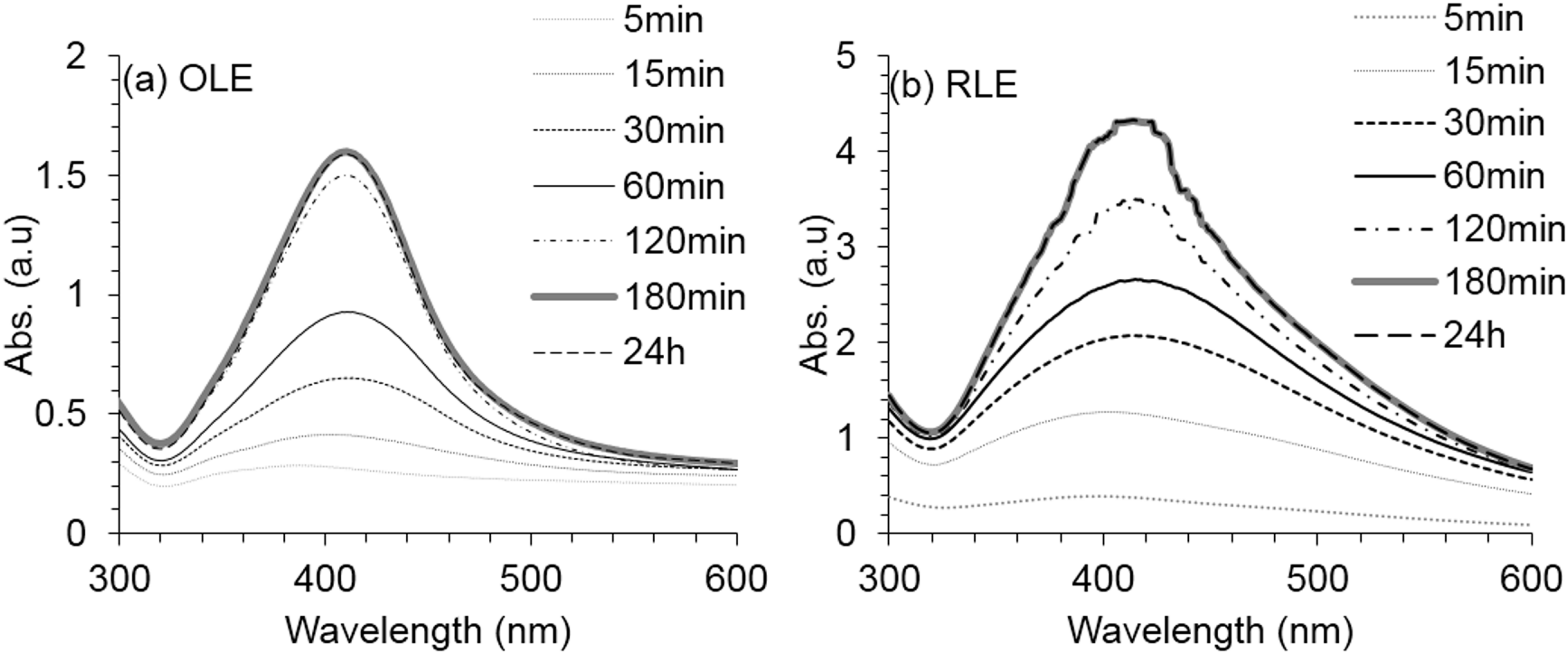
Average UV–vis spectra for AgNPs synthesis at different reaction time using (A) OLE and (B) RLE. Synthesis reaction conditions are fixed at PLE concentration of 250 mg/l; temperature of 80 °C and pH = 7 ± 0.2. Measurements were performed in triplicates.

The time for AgNP formation was also investigated for the best obtained OLE-AgNP and RLE-AgNP synthesis conditions. As shown in Fig. 4, for both nanoparticles, the peak intensity and spectral stability demonstrated that the reduction of silver ions and the formation of stable AgNPs was approximately completed within 3 h. After reduction completion, the absorption peak was found at a wavelength of 410 nm, which implied a AgNP core size of 69 nm and represented the highest production of AgNPs (Huang & Xu, 2010). These formation times are very rapid in comparison with some previously reported plant-mediated synthesis routes with reaction times of 24 h (Chandran et al., 2006) and 4 h (Vilchis-Nestor et al., 2008).

### AgNP characterization

At favorable conditions, the nanosuspensions were analyzed for AgNP concentration by AAS. The measured concentrations for OLE-AgNPs and RLE-AgNPs were approximately 50 and 45 mg/l, respectively. The percentages of Ag^+^ conversion into AgNP (Ag^0^) for OLE-AgNPs and RLE-AgNPs were 53% and 48%, respectively.

**Figure 5:**
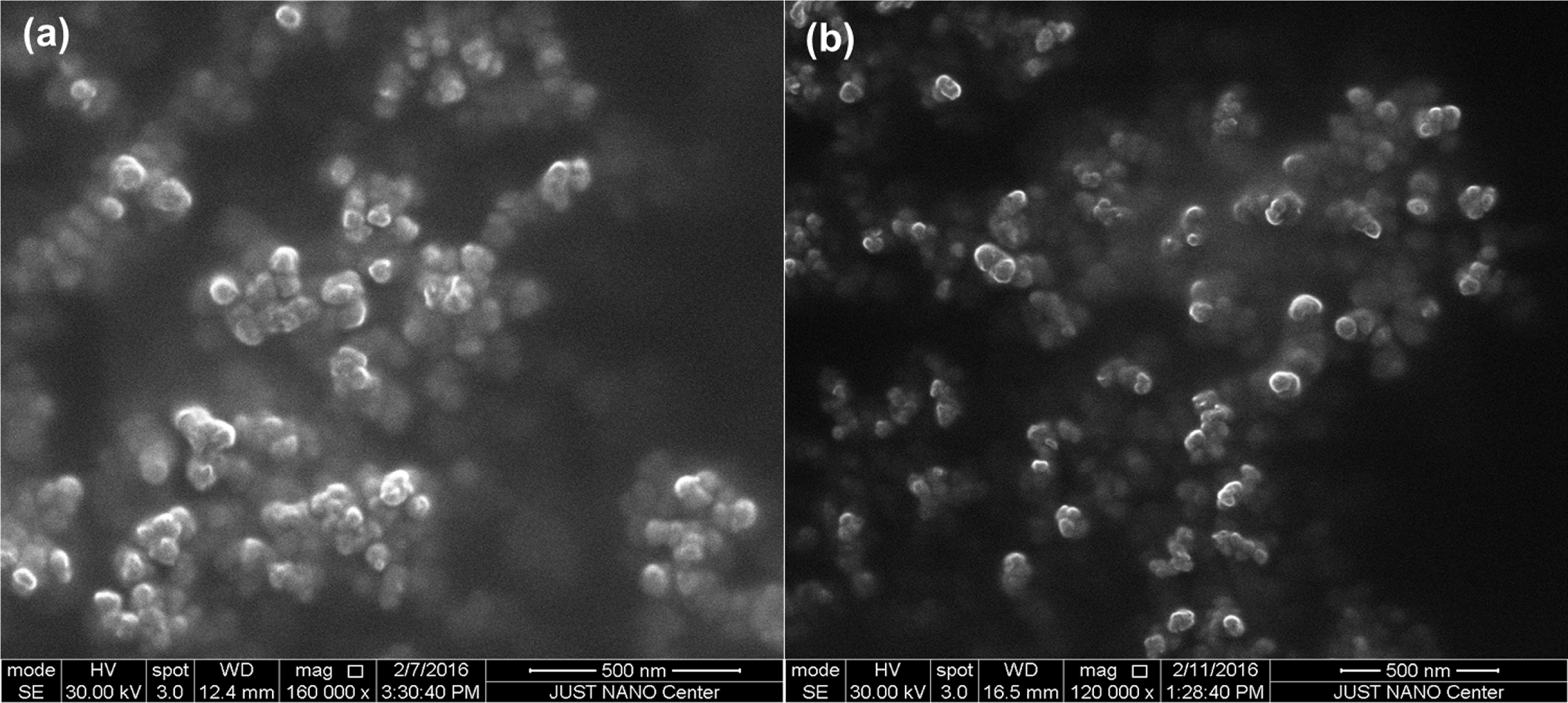
SEM micrographs of the synthesized AgNPs at the optimum conditions (A) OLE-AgNPS and (B) RLE-AgNPs. Synthesis reaction conditions are PLE concentration of 250 mg/l; temperature of 80 °C and pH = 7 ± 0.2.

**Figure 6:**
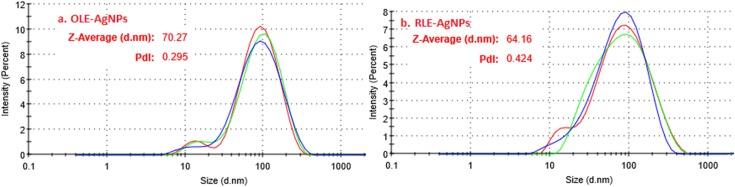
Intensity-hydrodynamic size distribution for (A) OLE-AgNPS and (B) RLE-AgNPs as obtained from the DLS.

The morphology of synthesized AgNPs by PLE was examined using ESEM. The ESEM images of AgNPs showed spherical particles with the average core sizes of 45 ± 2 and 38 ± 3 nm for OLE-AgNPs and RLE-AgNPs, respectively (Fig. 5). DLS measurements were performed to determine the average hydrodynamic size, size distribution, and PDI of the AgNPs. The particle size distribution curves of three consecutive measurements for both OLE-AgNPs and RLE-AgNPs are shown in Fig. 6. Measurement uncertainties are given as standard deviations. The average sizes (*Z*-average size) were 70.27 nm for OLE-AgNPs and 64.16 nm for RLE-AgNPs. The PDI was found to be 0.295 and 0.424 for OLE-AgNPS and RLE-AgNPs, which are both less than 0.7 indicating monodispersed particles (Honary et al., 2013). It was expected that the hydrodynamic diameter would be larger than the core because it includes surface coating materials and a solvent layer attached to the surface of the particle as it moves under the influence of Brownian motion (Hess, Frisch & Klein, 1986).

The AgNPs have negative zeta potentials (ζ) of −43.15 ± 3.65 mV for OLE-AgNPS and −33.65 ± 2.88 mV for RLE-AgNPs that indicate a high colloidal stability of the particles (O’Brien et al., 1990; El Badawy et al., 2010; Edison & Sethuraman, 2012). This negative zeta potential reflects the surface charge of negatively charged AgNPs after being functionalized with the extract compounds (Hunter, 2013). From the zeta potential value, it was evident that the synthesized nanoparticles were found to be stable using leaf extracts and the stability was even enhanced by adding PVP as a secondary capping agent.

### Fourier transform infrared spectroscopy

The dual action of leaf extracts as both reducing and capping agents was investigated using FTIR spectroscopy. In general, there are two regions in IR spectra: the functional group region (4,000–1,500 cm^−1^) and the fingerprint region (1,500–400 cm^−1^). The intense absorption peaks for both the extracts and the nanoparticles were used to evaluate the surface of the AgNPs produced. Because of the adsorption and modifications occurring at the surface, the functional group region is the most informative. Therefore, our analysis focused on that region for both the extracts and the synthesized AgNPs. The IR spectra of OLE and RLE show only minor differences (Table 2). RLE is mainly composed of isocarnosol (diterpene) dihydronormorphinone (alkaloid) and camphor (terpenoid) (Genena et al., 2008). The main chemical constituents of OLE are oleuropein, quercetin, rutin, and luteolin (all polyphenolic compounds) (Khalil, Ismail & El-Magdoub, 2012).

**Table 2:** FTIR analysis for OLE and RLE extracts.

Functional group	OLE IR peak (cm^−1^)	RLE IR peak (cm^−1^)
Amine N–H stretching	3,455	3,407
	3,311	3,282
O–H stretching (H-bonding)	3,187	3,223
C–H of alkanes	2,919	2,917
	2,848	2,849
C=O	1,728	1,727
Amide C=O stretch	1,688	1,685
C=C	1,688	1,685
N–H	1,608	1,606
C–O	1,515	1,516

**Figure 7:**
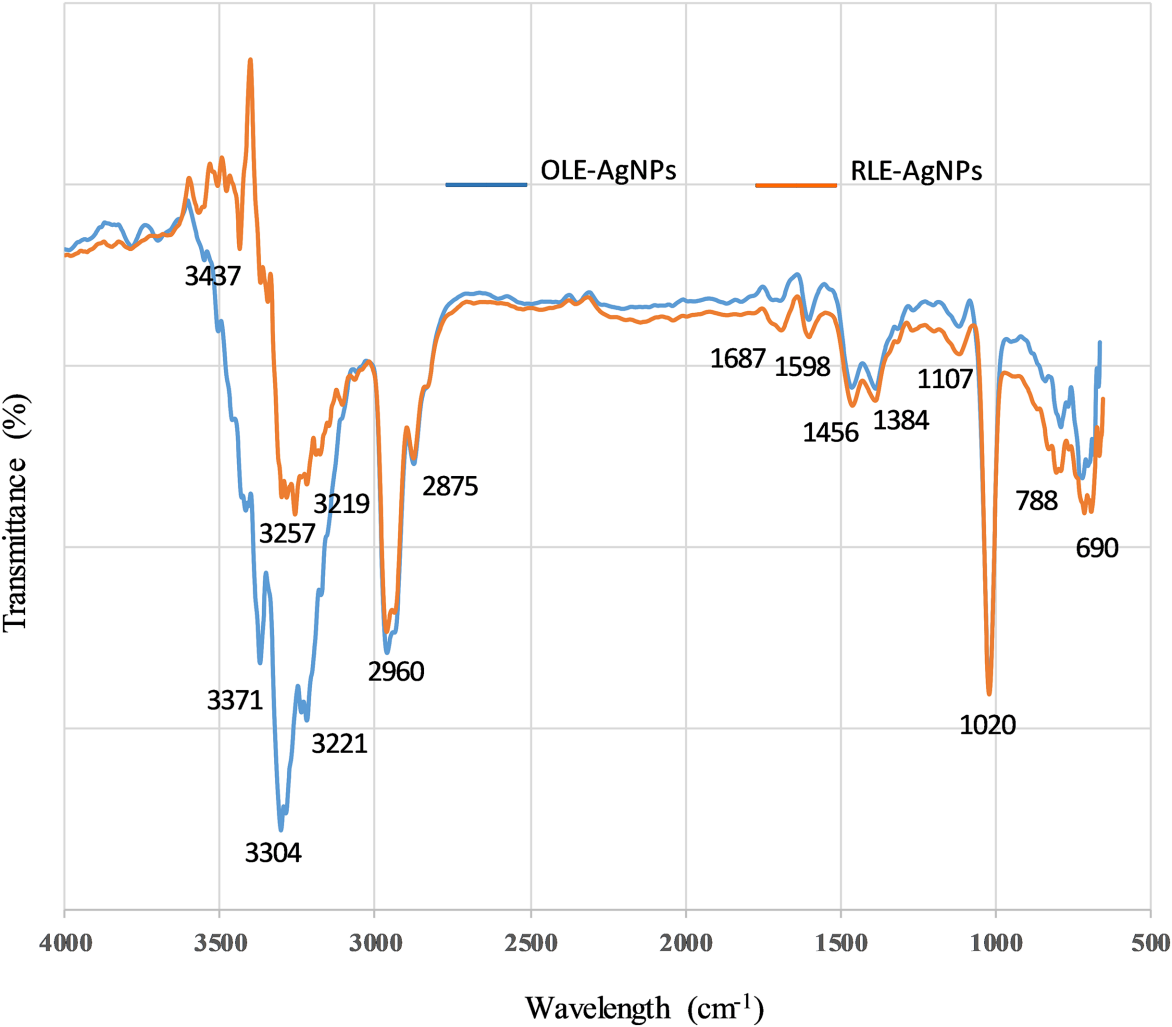
FTIR spectra of capped AgNPs with ______ OLE and with ______ RLE.

The spectra for both RLE-AgNPs and OLE-AgNPs (Fig. 7) were also similar to each other, as well as to the IR peaks of the plant extracts used for their synthesis. This is strong evidence that compounds present in the extracts not only participated in the reduction of silver ions, but also were adsorbed on the surface of the nanoparticles produced. The OLE-AgNPs show the presence of the following peaks 3,371, 3,304, 3,238, 3,221, 2,960, 2,875, 1,687, 1,598, 1,456, and 1,384 cm^−1^ at the functional group region, and the following peaks 3,437, 3,257, 3,219, 2,960, 2,887, 1,727, 1,687, 1,598, and 1,456 cm^−1^ were present in the IR spectrum of the RLE-AgNPs. The peaks at ~3,437, 3,371, and 3,257 were due to the −NH stretching of amine or −OH stretching of alcohols and phenols, or bending and stretching of hydrogen-bonded alcohols and phenols in the leaf extract. This small shift is an indication of adsorption on the surface, especially for the C=O (more than 10 cm^−1^). Shanmugam et al. (2014) suggested that these bonds could be due to the stretching of −OH in proteins, enzymes, or polysaccharides present in the extract. In addition, the peak at 2,966 cm^−1^ is due to C–H stretching and indicated the presence of alkanes, while the peaks at (2,930, 2,875) and (1,687), and (690) cm^−1^ corresponded to C–H stretching, C=C bond aromatic, and C–H bending respectively, and implied the presence of aromatic compounds. The peak at 1,687 cm^−1^ also could correspond to amide C=O stretching. The observed O–H and C=O modes for the OH and C=O groups might be attributed to oleuropein, apigenin-7-glucoside and/or luteolin-7-glucoside which, as suggested by Khalil, Ismail & El-Magdoub (2012), are flavonoid compounds present in the olive leaf. It can be concluded from the FTIR that the presence of organic functional groups, such as alkanes, aromatic compounds, and amide linkages of protein and amine, played a major role in the production and stability of AgNPs.

### Antimicrobial effect

To explore the antibacterial activity of OLE- and RLE-AgNPs, both the broth microdilution method (as recommended by CLSI protocol) and the Kirby–Bauer method were used. The MIC and MBC of OLE- and RLE-AgNP values against selected bacteria are listed in Table 3.

**Table 3:** Antibacterial activity of OLE-AgNPs and RLE-AgNPs against *S. aureus*, *Salmonella*, and *E-coli*.

Pathogenic bacteria	OLE-AgNPs		RLE-AgNPs	
	MIC μl/ml	MBC μl/ml	MIC μl/ml	MBC μl/ml
*Staphylococcus aureus* ATCC 25923	9.38	12.5	4.69	6.25
*Escherichia coli* ATCC 12900	9.38	12.5	4.69	6.25
*Salmonella enterica* CIP 104220	18.75	25	18.75	25

Table 3 shows that the OLE- and RLE-AgNPs exhibited good bactericidal activity against the three tested bacterial strains after 24 h of incubation. The MIC results for OLE-AgNPs when used against *Staphylococcus aureus* and *Escherichia coli* bacteria were 9.38 μl/ml, with MBC of 12.5 μl/ml. The MIC and MBC against *Salmonella* were 18.75 and 25 μl/ml, respectively. On the other hand, the MIC results for RLE-AgNPs against *Staphylococcus aureus* and *Escherichia coli* bacteria were 4.69 μl/ml, with MBC of 6.25 μl/ml; against *Salmonella* the MIC was 18.75 μl/ml and MBC of 25 μl/ml.

In the Kirby–Bauer method, silver nitrate was chosen as a positive control because of its well-documented antibacterial effects (Liau et al., 1997). The ZI in the antibacterial susceptibility experiments showed that OLE- and RLE-AgNPs have an inhibitory effect toward both gram-negative (*Escherichia coli* and *Salmonella*) and gram-positive (*Staphylococcus aureus*) bacteria, as shown in Table 4. The results showed that the synthesized AgNPs provided inhibition comparable to the control solution of AgNO_3_. The PLEs had a negligible inhibitory effect, most likely due to the low concentrations used (Ahmed et al., 2016a).

**Table 4:** Inhibition zones (IZ) in millimeters after treatment of bacteria with AgNO_3_ and PLEs using the Kirby–Bauer method.

Bacteria\Treatment	AgNO_3_ (170 mg/l)	OLE-AgNPs (50mg/l)	RLE-AgNPs (45mg/l)
*S. aureus*	18 ± 0.6 mm	13 ± 0.9 mm	12 ± 0.4 mm
*S. enterica*	20 ± 0.9 mm	12 ± 0.1 mm	8 ± 0.6 mm
*E. coli*	21 ± 1.1 mm	9 ± 0.3 mm	10 ± 0.6 mm

Silver (or what we now know as silver ions Ag^+^) was well-known for its antimicrobial properties even during ancient times (Sharma, Yngard & Lin, 2009). Zero-valent silver Ag^0^ (AgNPs) slowly releases silver ions via oxidation under aerobic conditions (Xiu, Ma & Alvarez, 2011). For this reason, the Kirby–Bauer method was selected, since its protocol is suitable for maintaining AgNP oxidation, and Ag^+^ is continuously released.

Table 5 compares our synthesis approach with some previously reported biological routes of AgNP synthesis using different natural plant extracts. As can be observed, most of the AgNPs were spherical in shape, in the range of 5–500 nm, with varied antibacterial potency. Huang et al. (2011), for example, studied the synthesis, formation mechanism, and antibacterial activity of biogenic AgNPs by Cacumen Platycladi Extract. They found that the MIC and MBC against *Escherichia coli* were 1.4 and 27 μl/ml, respectively, while the MIC against *Staphylococcus aureus* was 5.4 μl/ml. Our MBC results, however, were better than those reported by Huang et al. (2011), while their MIC results were better than ours. In addition, Tippayawat et al. (2016) also studied the antimicrobial activity of AgNP produced with Aloe vera plant extracts: the MIC of their AgNPs against gram-positive *Staphylococcus epidermidis* was 10 μl/ml, which is comparable with our MIC results. However, their AgNPs were fabricated at a higher temperature (100 °C for 6 h and 200 °C for 12 h).

**Table 5:** Synthesis of silver nanoparticles and their antimicrobial activity using previous reported plant extracts as compared to Tollens’ method and current study.

Plant leaf extracts/saccharides	Average size (nm)	Zeta potential (ζ) mV	Antimicrobial activity	Reference
*Cinnamon zeylanicum*	Spherical 31–40 nm (TEM)	Negative zeta potential	Growth inhibition study MIC was 50 μl/ml and EC_50_ of 11 ± 1.72 μl/ml against *E-coli* strain BL-21	Sathishkumar et al. (2009)
*Mentha piperita*	90 nm (SEM)	NA	Well diffusion method The antibacterial activity of silver nanoparticles against *E. coli* was higher than that against *S. aureus*	Mubarak Ali et al. (2011)
Cacumen Platycladi	Uniform spheroidal 18.4 ± 4.6 nm (TEM)	NA	Agar well diffusion method and broth medium methods MIC and MBC were 1.4 and 27 μl/ml against *E. coli*. MIC was 5.4 μl/ml against *S. aureus*	Huang et al. (2011)
Mangosteen	35 nm (TEM)	NA	Disk diffusion method using 20 μg/ml AgNPs *IZ was 15 mm against *E. coli* and 20 mm against *S. aureus*	Veerasamy et al. (2011)
*Rosmarinus Officinalis*	Stable particles 60 nm (XRD)	NA	Agar well diffusion method using two mM AgNPs *IZ was 25 mm against *S. aureus*, 24 mm against *S. pneumoniae,* 24 mm against *C. albicans*, and 22 mm against *E. coli*, *K. pneumonia*, *P. aeruginosa*, and *Proteus volgaris*	Sulaiman et al. (2013)
Olive	Mostly spherical 20–25 nm (TEM)	NA	Agar well diffusion method The AgNPs at 0.03–0.07 mg/ml concentration significantly inhibited bacterial growth against *S. aureus*, *P. aeruginosa* and *E. coli*	Khalil et al. (2013)
Lantana camara	20 nm nearly spherical (FESEM and TEM)	−36	Agar well diffusion method using 0.001M AgNPs *IZ was three to seven mm against Bacillus spp, *Pseudomonas* spp, *Staphylococcus* spp, and *E. coli.*	Ajitha et al. (2015)
Aloe vera	70.7–192.02 nm (XRD–SEM)	NA	Agar well diffusion method using 0.1 mg/ml of AgNPs *IZ was 1.5–3.9 cm against *S. epidermidis*, and 1.4–3.9 cm against *P. aeruginosa* Microdilution method: MIC was 10 μl/ml against *S. epidermidis*	Tippayawat et al. (2016)
Olive	Spherical and stable 45 ± 1.53 nm (SEM)	−43.15 ± 3.65 (DLS)	Agar well diffusion method using 50 μg/ml of AgNPs *IZ were 9, 13, and 12 mm for *E-coli, S. aureus and S. enterica,* respectively Microdilution method: MIC was 9.38 μl/ml against *E-coli* and *S. aureus,* and 18.75 μl/ml against *S. enterica*	This study
Rosemary	Spherical and stable 38 ± 2.71 (SEM)	−33.65 ± 2.88 (DLS)	Agar well diffusion method using 45 μg/ml of AgNPs *IZ were 10,12, and 8 mm for *E-coli, S. aureus, and S. enterica,* respectively Microdilution method: MIC was 4.69 μl/ml againt *E-coli* and *S. aureus; and 18.75 μl/ml against S. enterica*	This study
Tollens’ method	25–100 nm with narrow size distributions	NA	Standard dilution micro method MIC and MBC were 13.5, 27, 3.38, and 13.5 μg/ml against *Pseudomonas aeruginosa* using 108 μg/ml AgNPs prepared by glucose, galactose, maltose, and lactose, respectively	Panáček et al. (2006)

**Note:**

* IZ, Inhibition Zone.

These results offer insights into the potential of scaling up the production of AgNPs using leaf extracts from olive and rosemary plants for commercial implementation.

The antimicrobial mechanisms of AgNPs are still not completely understood. One mechanism proposed is that AgNPs are able to interact with the bacterial cell wall, alter its properties via decaying lipopolysaccharide molecules, and form “pits” that increase wall permeability (Sondi & Salopek-Sondi, 2004). In addition, antibacterial properties have been reported to be size-dependent, with higher antimicrobial performance at smaller sizes of nanoparticles (Ajitha et al., 2015; Martinez-Castanon et al., 2008).

## Conclusions

In conclusion, AgNPs were successfully manufactured through the reduction of Tollens’ reagent in conjunction with OLE and RLE. Rapid formation time of AgNPs was achieved in approximately 3 h. The synthesis conditions such as extract concentration, temperature, and pH highly affected the synthesis. We were able to optimize the synthesis to obtain a smaller AgNP core size, an approximately spherical shape, and stability. The functional groups present in the RLE-AgNPs and OLE-AgNPs played a major role in the production and stability of AgNPs.

The results showed that the synthesized AgNPs provided inhibition properties comparable to the control solution of AgNO_3_, and better or equal to other reported biosynthesis approaches. Finally, further investigations are recommended to analyze the antimicrobial mechanisms and to explore the potential of scaling up the proposed methodology.

## Supplemental Information

10.7717/peerj.6413/supp-1 Supplemental Information 1 UV-Vis absorbance data.

10.7717/peerj.6413/supp-2 Supplemental Information 2 FTIR raw data results for plant extracts (OLE and RLE).

10.7717/peerj.6413/supp-3 Supplemental Information 3 FTIR raw data results for synthesized silver nanoparticles (OLE_AgNPs and RLE_AgNPs).

10.7717/peerj.6413/supp-4 Supplemental Information 4 Photos of some Agar plates that were treated with AgNO_3_and the synthesized AgNPs.

## Additional Information and Declarations

### Data Availability

The following information was supplied regarding data availability: The raw data are available in the Supplemental Files.
