# Supplementary material for: Synthesis of silver nanoparticles using a modified Tollens’ method in conjunction with phytochemicals and assessment of their antimicrobial activity
Source: PeerJ. 2019 Feb 8;7:e6413. doi: 10.7717/peerj.6413 (PMC6369825; doi:10.7717/peerj.6413)
Supplement: Supplemental Information 4 [file peerj-07-6413-s004.docx]

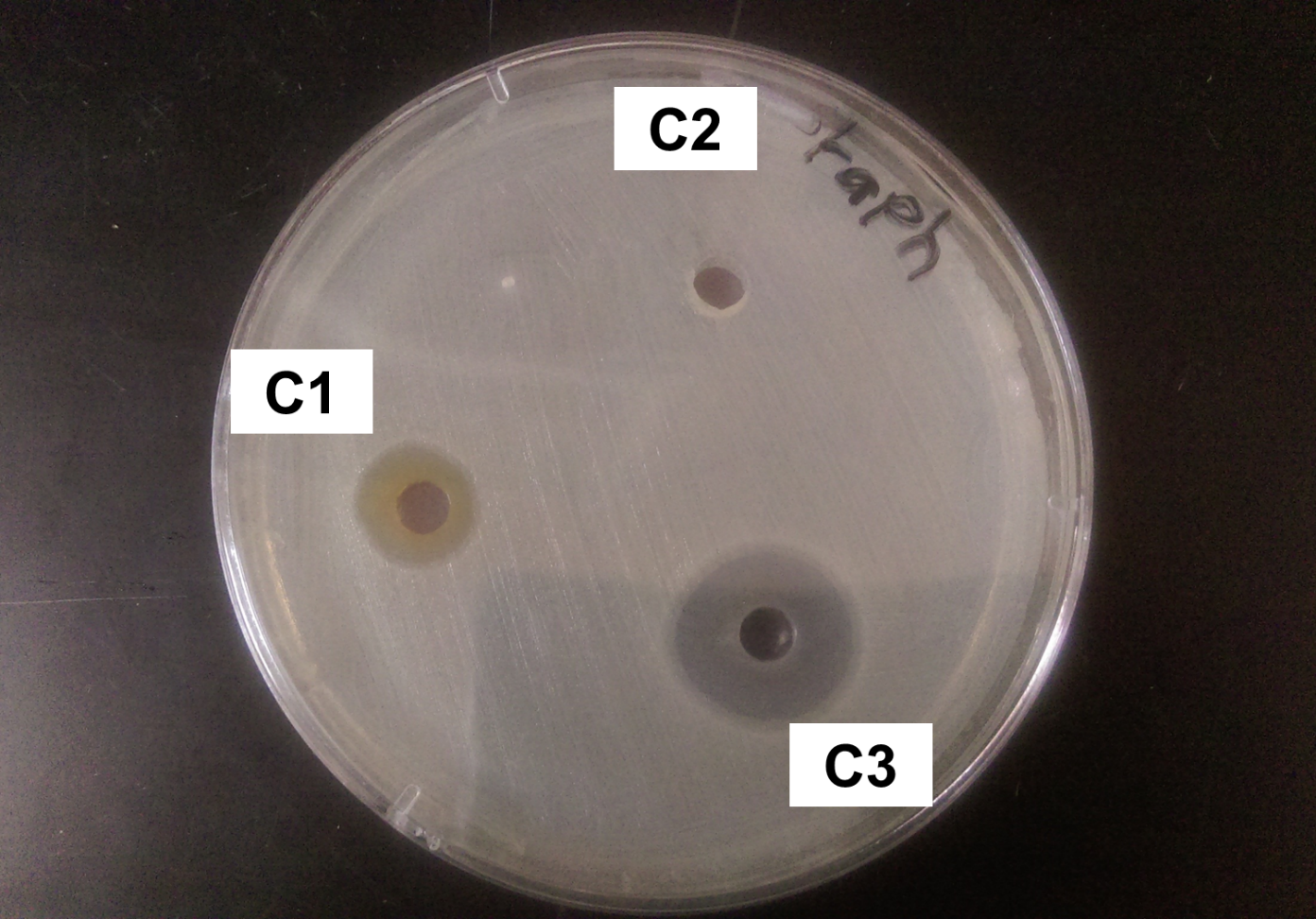


Figure 1: AgNO_3_ effect on (C3), AgNPS-OLE (C3) and OLE alone effect on Staphylococcus aureus.


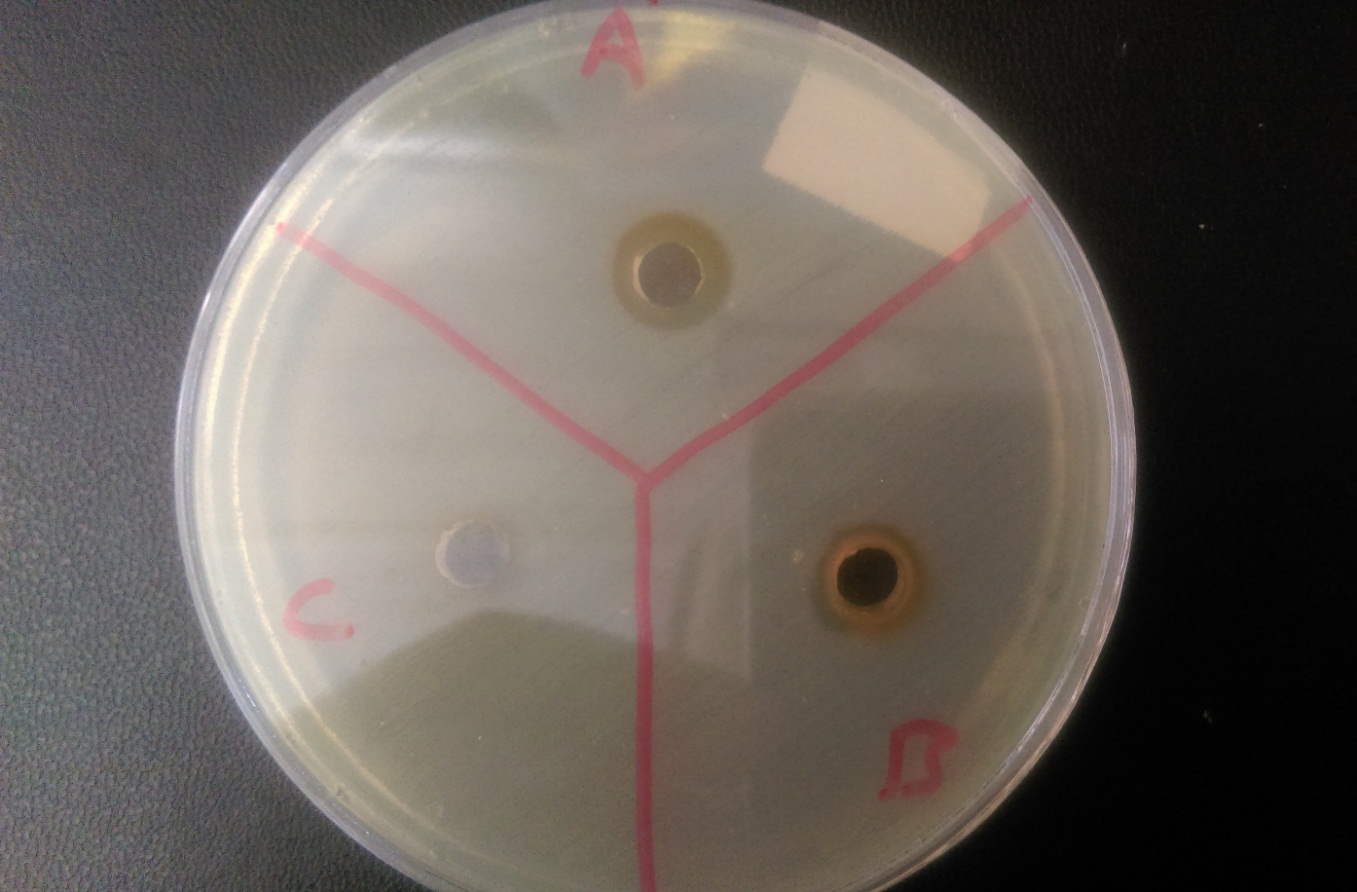


Figure 2: (A) and (B) AgNPs-OLE effect on E-Coli. (C) is a control of only the OLE.


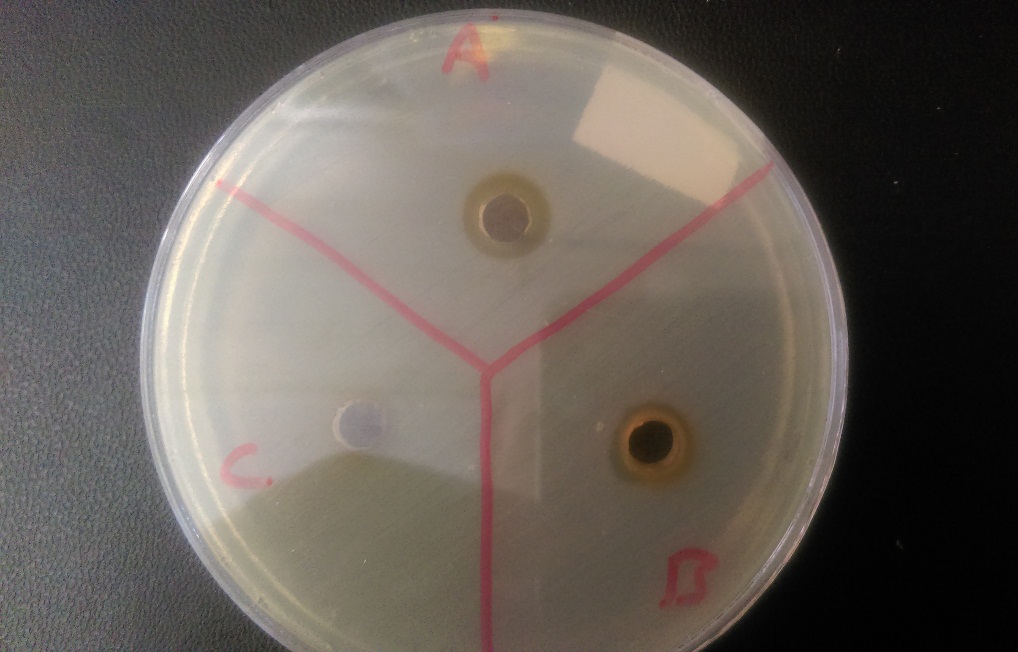


Figure 3: (A) and (B) AgNPs-OLE effect on Salmonella. (C) is a control of only the OLE.


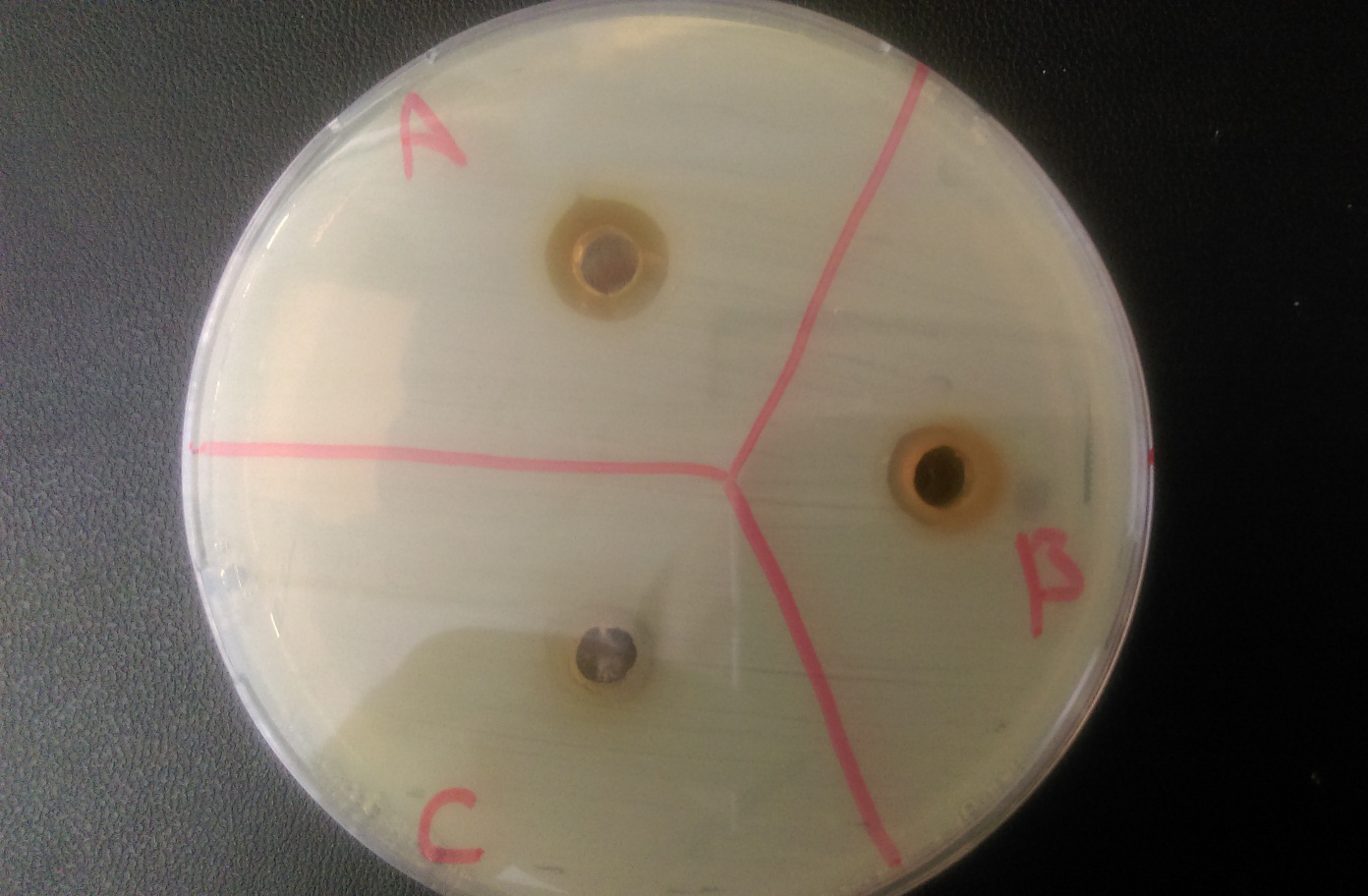


Figure 4: (A) and (B) AgNPs-OLE effect on Staphylcoccus aureus. (C) is a control of only the OLE.


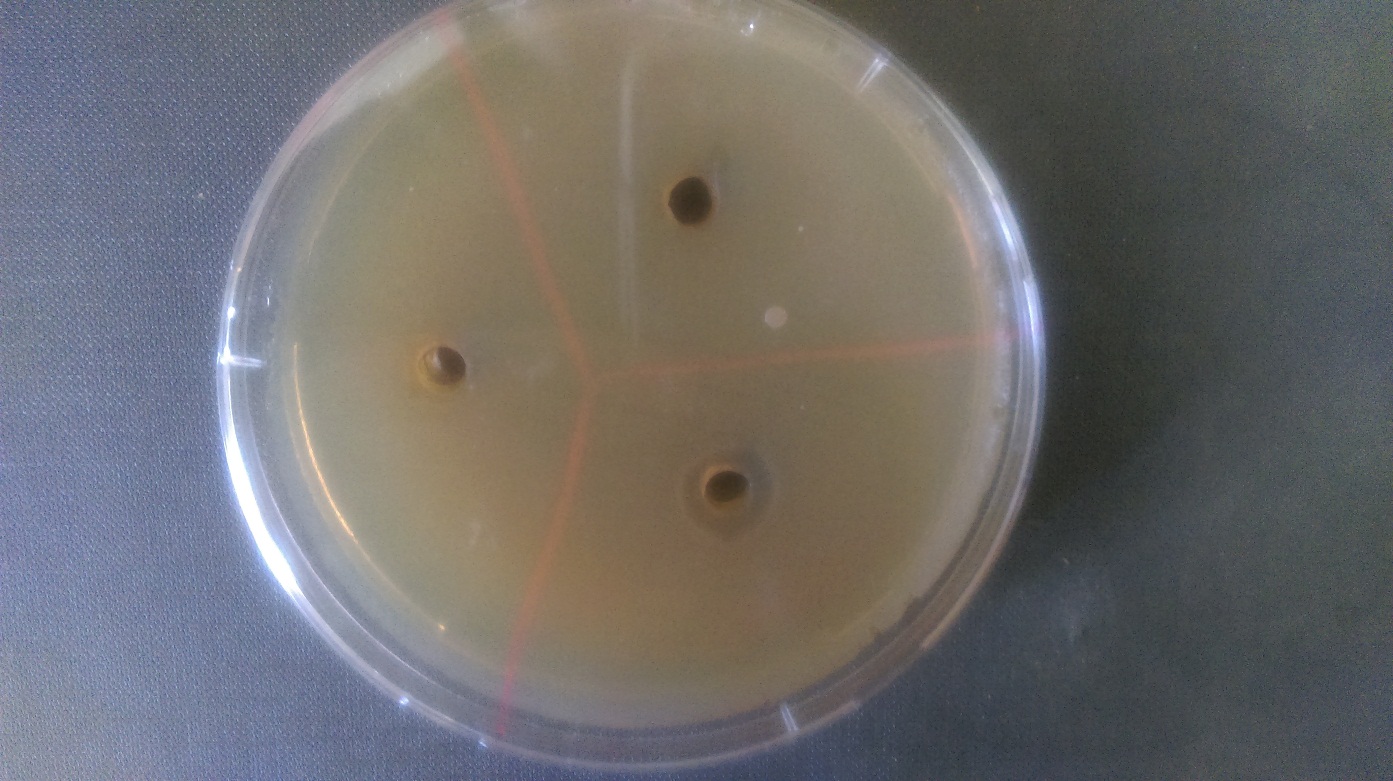


Figure 5: AgNPs-RLE effect on E-Coli, the well with the circle surrounding it. The others are controls of RLE only.


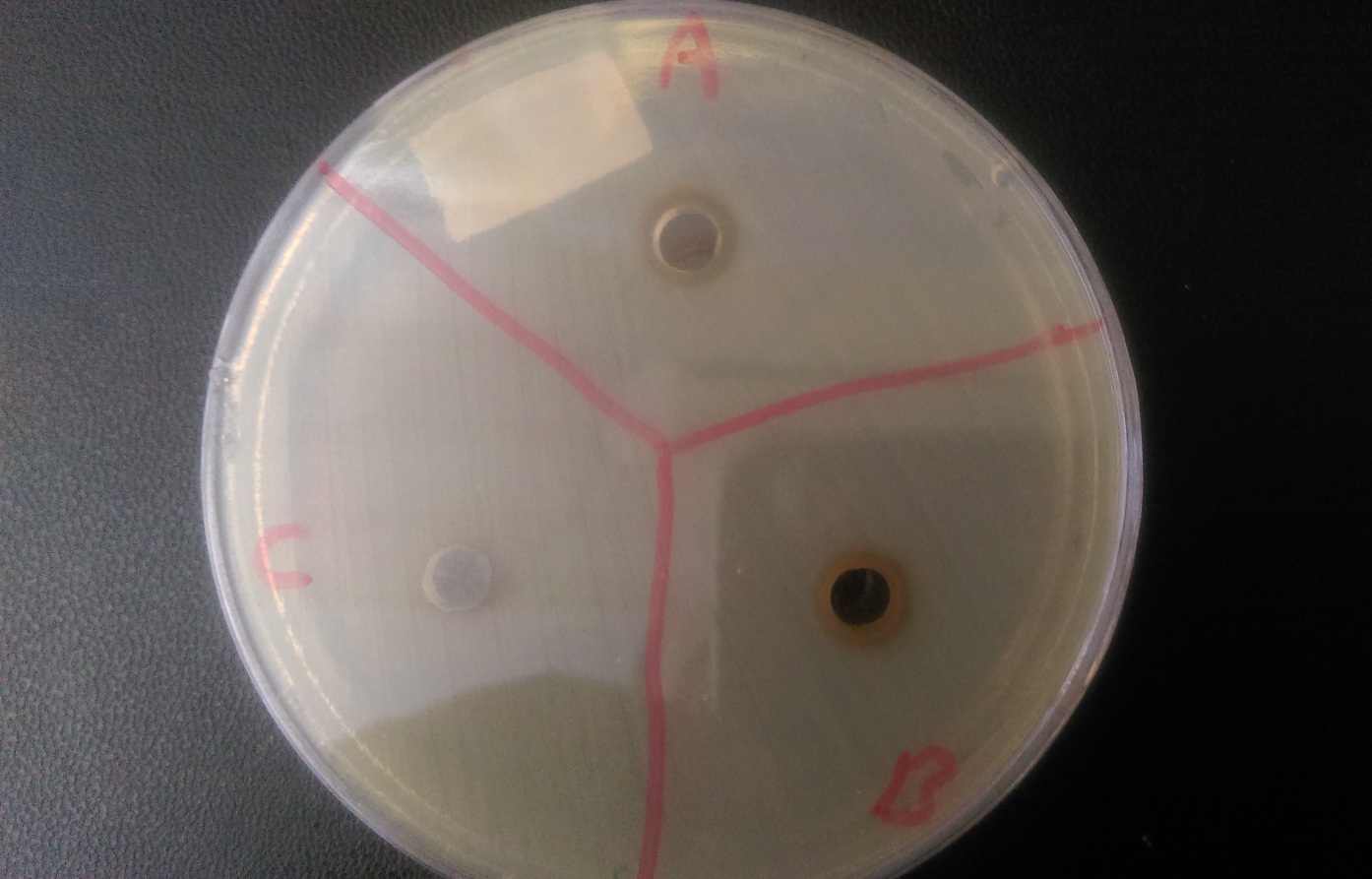


Figure 6: (A) and (B) AgNPs-RLE effect on Salmonella. (C) is a control of only the RLE.


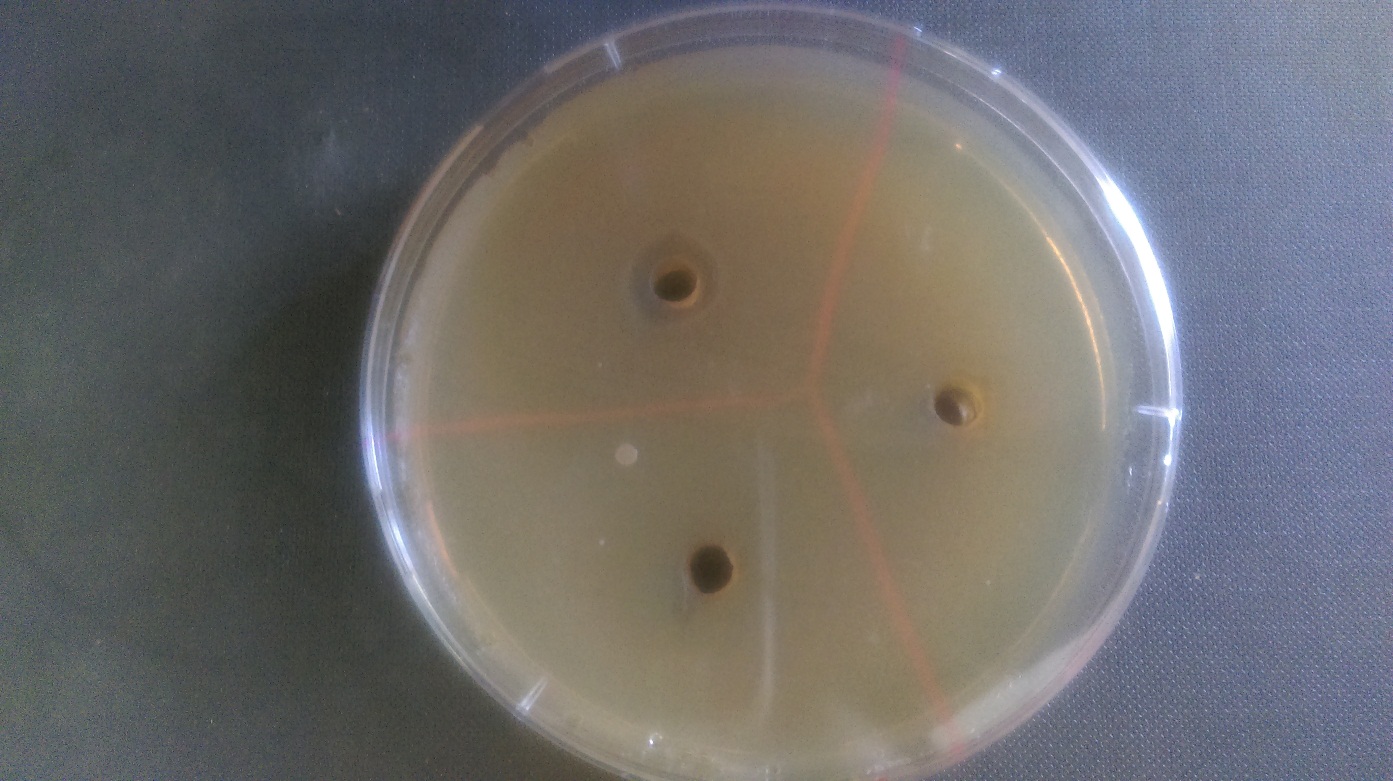


Figure 7: (A) and (B) AgNPs-OLE effect on Staphylcoccus aureus, the well with the circle surrounding it. The others are controls of OLE only.
